# Comprehensive Expression Analyses of Plastidial Thioredoxins of *Arabidopsis thaliana* Indicate a Main Role of Thioredoxin *m2* in Roots

**DOI:** 10.3390/antiox11071365

**Published:** 2022-07-14

**Authors:** Mariam Sahrawy, Juan Fernández-Trijueque, Paola Vargas, Antonio J. Serrato

**Affiliations:** Departamento de Bioquímica, Biología Celular y Molecular de Plantas, Estación Experimental del Zaidín, Consejo Superior de Investigaciones Científicas (CSIC), 18008 Granada, Spain; sahrawy@eez.csic.es (M.S.); trijuek@gmail.com (J.F.-T.); paola.vargas@eez.csic.es (P.V.)

**Keywords:** thioredoxin, redox, GFP, plastid, fructose-1,6-bisphosphatase

## Abstract

Thioredoxins (TRXs) *f* and *m* are redox proteins that regulate key chloroplast processes. The existence of several isoforms of TRXs *f* and *m* indicates that these redox players have followed a specialization process throughout evolution. Current research efforts are focused on discerning the signalling role of the different TRX types and their isoforms in chloroplasts. Nonetheless, little is known about their function in non-photosynthetic plastids. For this purpose, we have carried out comprehensive expression analyses by using *Arabidopsis thaliana* *TRXf* (*f1* and *f2*) and *TRXm* (*m1, m2, m3* and *m4*) genes translationally fused to the green fluorescence protein (GFP). These analyses showed that TRX *m* has different localisation patterns inside chloroplasts, together with a putative dual subcellular localisation of TRX *f1*. Apart from mesophyll cells, these TRXs were also observed in reproductive organs, stomatal guard cells and roots. We also investigated whether photosynthesis, stomatal density and aperture or root structure were affected in the TRXs *f* and *m* loss-of-function Arabidopsis mutants. Remarkably, we immunodetected TRX *m2* and the Calvin–Benson cycle fructose-1,6-bisphosphatase (cFBP1) in roots. After carrying out in vitro redox activation assays of cFBP1 by plastid TRXs, we propose that cFBP1 might be activated by TRX *m2* in root plastids.

## 1. Introduction

Thioredoxins (TRXs) are characterized by the high number of isoforms found in chloroplasts, named *f*, *m*, *x*, *y* and *z*; a size of about 12 kDa; and the conserved active site WC(G/P)PC. This redox motif is involved in the post-translational modification of target proteins through the reduction of disulphide bonds [1,2]. The ferredoxin thioredoxin system (FTS), which includes ferredoxin (Fdx) and ferredoxin thioredoxin reductase (FTR), allows the reduction of TRXs in chloroplasts. For many years, the best described chloroplast TRXs were TRXs *f* and *m*. One of the most important biological processes in chloroplasts, the Calvin–Benson cycle (CBC), is controlled by TRX *f*, and one of its best-known targets is the CBC fructose-1,6-bisphosphatase (cFBP1) [3]. In addition, *m*-type TRXs, originally described as reducers of the malate dehydrogenase (MDH), are more related to photosynthesis [4]. The *Arabidopsis thaliana* genome contains two TRX *f* proteins (TRX *f1* and *f2*), four TRX *m* proteins (TRX *m1*, *m2*, *m3* and *m4*), two TRX *y* proteins (TRX *y1* and *y2*), one TRX *x* and one TRX *z* [5,6]. TRX *f* proteins are of eukaryotic origin, whereas *m*-type TRXs are of prokaryotic origin [7]. Several studies have indicated a wide range of different functions in plant processes [8,9,10,11,12], and it is not evident whether TRX diversity suggests functional redundancy or specificity for target proteins in plants. It has been known for several years that chloroplast TRXs regulate processes such as starch metabolism, oxidative stress response, lipid biosynthesis, nitrogen metabolism, protein folding, and translation [13,14,15]. An interesting study by Barajas and colleagues [16] showed evidence of the presence, in heterotrophic tissues, such as roots or flowers, of plastidial TRXs *f* and *m* of *Pisum sativum*, supporting new roles for these proteins. Even more curious was the isolation in wheat endosperm of amyloplasts of a complete ferredoxin–TRX system, which includes Fdx, Fdx-NADP^+^ reductase (FNR) and FTR [17]. One of the most important processes that occurs in chloroplasts is photosynthesis, which generates ATP and NADPH, used, among other metabolic pathways, for CO_2_ fixation and carbohydrate synthesis. In this regard, stomata play an important role by regulating gas exchange (CO_2_ and H_2_O) and modulating photosynthesis in function of environmental factors such as air flow, temperature or humidity [18]. On the other hand, roots perform an essential function by taking water and nutrients from the soil and transporting them to other parts of the plant, as leaves, in a coordinated manner. Besides, oxygen and reactive oxygen species (ROS) are among the most important signalling molecules that participate in most of the processes that take place in plants and that allow long distance communications between organs from apical meristems to roots [19]. ROS level and the redox state of the proteins involved in the growth and development of the roots are under the control of redox enzymes such as TRXs, which are present in the cytosol, chloroplasts, mitochondria and the nucleus [20].

Chloroplast proteins related to photosynthesis are usually investigated in green tissues. However, the study of these proteins in non-photosynthetic (or partially photosynthetic) organs is usually neglected. Assuming that a part of plastid metabolism and regulation might be also operating in non-green tissues, chloroplast TRXs would be expressed in these tissues as well. Nevertheless, predicting protein expression through promoter analyses is not always useful, as protein expression can be under post-transcriptional or translational regulation. In addition, experimental approaches such as qRT-PCR or Western blot analyses can give us valuable quantitative expression levels, but do not provide any information regarding tissue specificity. Instead, protein expression can be followed *in planta* with fluorescence microscopy, through the analysis of plant lines bearing genes translationally fused to the green fluorescent protein (GFP). This experimental approach, highly sensitive, would be of particular interest for the study of protein expression in plant multigenic families, such as the TRX family.

With the aim of having a broad outlook on the function of the plastid TRXs *f* and *m* in plants, we have carried out a comprehensive expression analysis of six TRX(*f*/*m*)::GFP lines in the model plant *A. thaliana*. Our study has shown that TRX *m* has different sub-chloroplast localizations. Differences in photosynthesis, transpiration and stomatal conductance, besides the stomata aperture, suggest possible functional specificity among the TRXs. Interestingly, according to our results, *A. thaliana* TRX *m2* is probably one of the most expressed TRXs in plastid roots, highlighting the importance of redox signalling mediated by TRXs in the non-photosynthetic plastids. Comparative TRX activation assays of cFBP1, a TRX target that we also detected in roots, led us to propose that TRX *m2* might be an alternative cFBP1 activator in the plastid roots of Arabidopsis.

## 2. Materials and Methods

### 2.1. Arabidopsis Plant Growth Conditions

Wild type lines (ecotypes Columbia-0 (Col0) and *Landsberg erecta* (Ler)) and TRX-defective mutants *trxf1*, *trxf2*, *trxm1*, *trxm2*, *trxm4* (genetic background Col0) and *trxm3* (genetic background Ler) [12,21] of *A. thaliana* were grown in 0.5× MS medium supplemented with 0.5% sucrose containing 0.8% agarose for root analyses (for confocal and light microscopy analyses and Western blotting assays). In vitro plants were cultured in a growth chamber at 22 °C/21 °C (light/dark) under long-day conditions (16-h-light/8-h-dark) and with a photosynthetically active radiation of 100 µmol photons m^−2^ s^−1^. For cotyledon observations with a confocal microscope, 7-day-old Arabidopsis seedlings were used. Rosettes and roots from 21-day-old plants were sampled and immediately transferred to liquid nitrogen before storage at −80 °C for protein expression analysis.

For GFP analyses in reproductive organs, Arabidopsis GFP lines were planted in soil and grown in growth chambers under long-day conditions (16-h-light/8-h-dark) at 22 °C/21 °C (light/dark), 60% relative humidity, and with a photosynthetically active radiation of 120 µmol photons m^−2^ s^−1^. Plants were grown for 4 weeks before confocal observations.

### 2.2. Protein Extraction and Western Blot Analysis

For protein extraction, a pool of a minimum of 6 plants (rosette leaves and roots) were sampled to perform Western blotting analyses according to Serrato and co-workers (2018) [22].

### 2.3. GFP Fusion Constructs

Arabidopsis plants were transformed with pGWB4-derived constructions expressing the proteins, translationally fused with GFP, of the different TRXs *f* and *m* [23]. Genes, including 1-kb promoters, were cloned into a pGWB4 vector by using the Gateway technology (Invitrogen, Life Technologies Corp., Carlsbad, CA, USA). A total of six constructions were obtained. Cloning oligos are shown in Table S1.

### 2.4. Confocal Microscopy Observation of GFP Fluorescence and Propidium Iodide Staining

For the detection of the fluorescence signal and the capture of high-resolution images in different organs, the Confocal Laser Scanning Microscopy Modular System C-1 (Nikon Instruments Europe B.V., Badhoevedorp, The Netherlands) was used [24]. For staining with propidium iodide (PI), 5-day-old roots were incubated in the dark for 10 min in a 10 µg/mL solution. Then, roots were washed with distilled water before microscope observation [25].

### 2.5. Scanning Electron Microscopy

By using the high-resolution scanning electron microscope (HRSEM) AURIGA (Carl Zeiss SMT, Oberkochen, Germany), the number, shape and opening of the stomata were observed and measured in the leaf adaxial side of at least 3 Arabidopsis mutant plants. High resolution and low voltage images of the secondary electron (SE), taken by a SE in-lens detector of 500-fold magnification, were analysed with the ImageJ 2.0.0 software (ImageJ, U. S. National Institutes of Health, Bethesda, MD, USA, https://imagej.nih.gov/ij/, 1997–2018, accessed on 12 July 2022) for stomata analysis.

### 2.6. Stomatal Conductance and Transpiration Determinations

The conductance and transpiration were determined by using a portable infrared gas analyser LI-6400 (LI-COR Biosciences, Inc., Lincoln, NE, USA), which allows the analysis of CO_2_ assimilation rate in the upper fully expanded leaf of the *A. thaliana* wild type (Col0) and *trxm* mutants under controlled environmental conditions. The data were fixed when the light intensity reached 2000 PAR (µmol photons m^−2^ s^−1^). The LI-6400 6.1 software was used to calculate the photosynthetic parameters.

### 2.7. In Vitro FBPase Activation Assays

Coding sequences for the mature proteins were cloned into the expression vector pET-28b, which adds a His-tag. Recombinant proteins were purified by Co^2+^ affinity chromatography (GE Healthcare Life Sciences, Marlborough, MA, USA) according to the manufacturer’s instructions. Oligonucleotides used for cloning are listed in Table S1.

For redox activation assays, 2.5 μM cFBP1 was incubated for 30 min at 22 °C with DTT (concentration depending on the assay) or 0.1 mM DTT and TRX (concentration depending on the assay) in the presence of 100 mM Tris-HCl (pH 8.0). Then, free thiols were alkylated by incubation in the dark for 30 min at 37 °C in the presence of equal volumes of a solution containing 60 mM iodoacetamide, 2% SDS, and 100 mM Tris-HCl pH 8. FBPase redox state was determined by Western blotting analyses as published by Serrato and co-workers (2018) [22].

After redox activation assays, FBPase activity (2 μg cFBP1) was determined as previously described by Rojas-González and co-workers (2015) [26]. The assay was performed in microtiter plates in a final volume of 200 μL, containing the following components: redox activated FBPase, 100 mM Tris-HCl buffer pH (8.0), 1 mM MgCl_2_, 0.6 mM fructose-1,6-bisphosphate, 0.3 mM NADP^+^, 0.7 U phosphoglucose isomerase (ROCHE), and 0.3 U glucose-6-phosphate dehydrogenase (ROCHE, Basel, Switzerland). The increase of absorbance at 340 nm (NADPH formation) versus time was read with a microplate reader Tecan Sunrise™ (Tecan Trading AG, Männedorf, Switzerland). Incubations and activity assays were performed at room temperature (22–24 °C).

### 2.8. Statistical Analyses

Statistical analyses of data were performed using R software (RStudio Team (2020). RStudio: Integrated Development for R. RStudio, PBC, Boston, MA, USA. Available online: http://www.rstudio.com/, accessed on 16 June 2022). Significant differences between groups were tested using the ANOVA test and the pair-wise comparison Tukey HSD test with the “Agricolae package” (agricolae: Statistical Procedures for Agricultural Research. R package version 1.4.0. Available online: https://myaseen208.github.io/agricolae/https://cran.r-project.org/package=agricolae, accessed on 16 June 2022).

## 3. Results

### 3.1. Location of TRXs f and m in Photosynthetic and Heterotrophic Tissues

**Figure 1:**
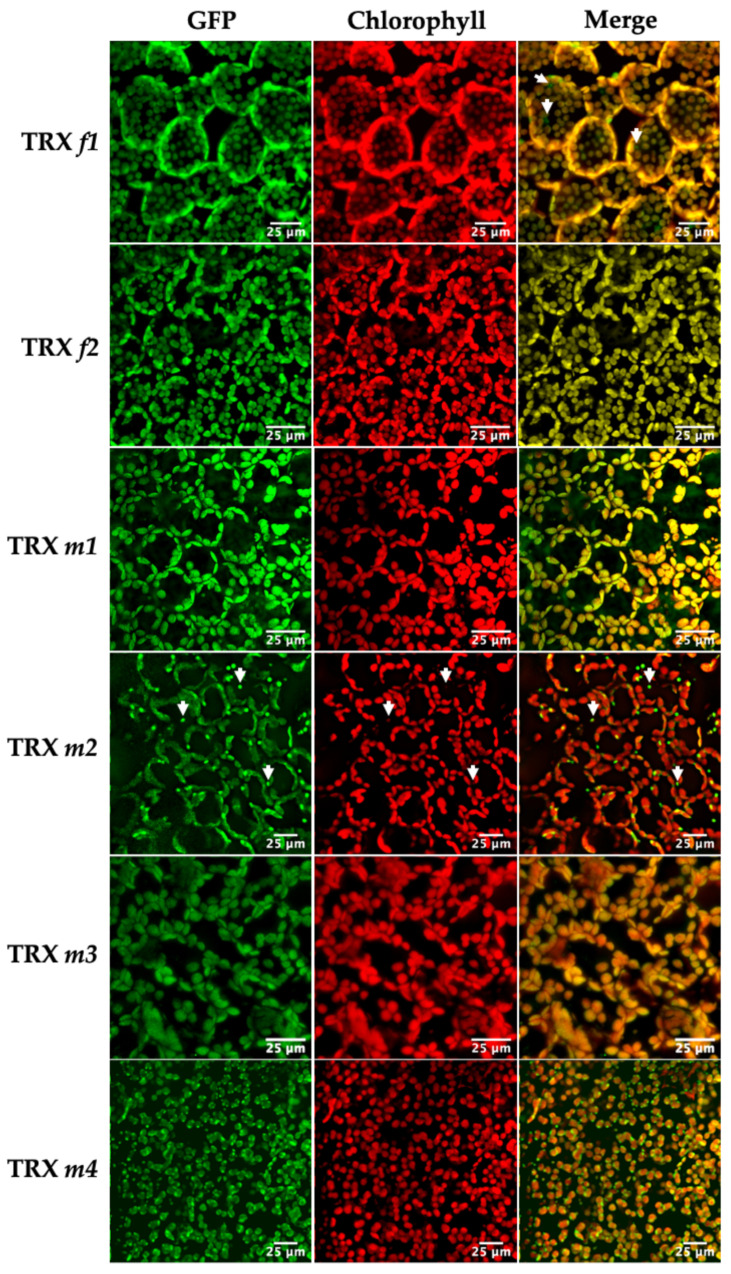
Representative confocal images of Arabidopsis lines expressing the TRX(*f/m*)::GFP constructs in the cotyledon mesophyll of 7-day-old Arabidopsis seedlings. GFP, fusion protein fluorescence; Chlorophyll, chlorophyll autofluorescence; Merge, the computer overlay of the two fluorescence images. Arrows highlight some peculiar sub-cellular localisations (explained in Results).

**Figure 2:**
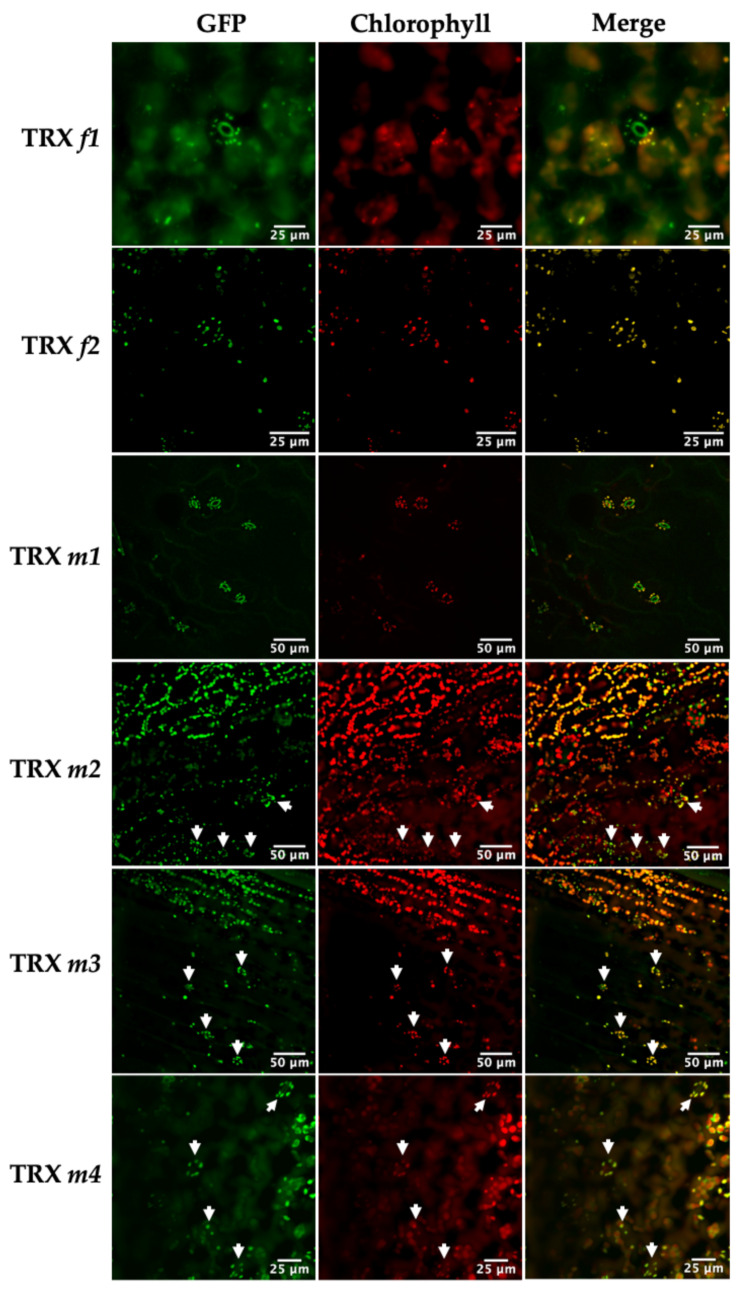
Representative confocal images of Arabidopsis lines expressing the TRX(*f/m*)::GFP constructs in stomata of 7-day-old Arabidopsis seedlings. GFP, fusion protein fluorescence; Chlorophyll, chlorophyll autofluorescence; Merge, the computer overlay of the two fluorescence images. Arrows highlight stomata localisation of the TRXs analysed.

The expression of the TRX*f1*::GFP and TRX*f2*::GFP constructs was observed in chloroplasts of the cotyledon mesophyll. Figure 1 clearly shows the chloroplast localization of both TRX *f* isoforms. However, TRX*f1*::GFP was also observed in sub-cellular compartments with no chlorophyll (arrows in Figure 1, TRX *f1*). This result suggests that TRX *f1* might have a role independent of photosynthesis or the Calvin–Benson cycle. Analyses of reproductive and heterotrophic organs revealed that *f*-type thioredoxins were restricted to green tissues. The presence of both isoforms was also found in chloroplasts of the stomatal guard cells in the cotyledon epidermis (Figure 2).

Confocal images also confirmed the presence of the *m*-type isoforms in chloroplasts (Figure 1). TRX*m1*::GFP showed the strongest GFP signal in chloroplasts, while TRX*m3*::GFP displayed the lowest one. It should be noted that TRX *m2* displayed a distinctive signal pattern (Figure 1) coming from vesicle-like structures, co-localised with chlorophyll fluorescence. Similar vesicles have been reported to appear in chloroplast autophagy [27]. Finally, TRX*m4*::GFP was distributed throughout the chloroplasts, but predominantly in uneven patterns around the periphery. A similar pattern was found for the protein thylakoid formation1 (Thf1), perhaps indicating an association of TRX *m4* with the chloroplast envelope as it was proposed for Thf1 [28]. Figure 2 displays TRXs *m*::GFP expression in stomatal guard cells of epidermal tissue. Regarding redox regulation, the presence of TRXs *f* and *m* is quite obvious in stomatal chloroplasts, probably meaning that these proteins might have an active role in stomata regulation during gas exchange.

**Figure 3:**
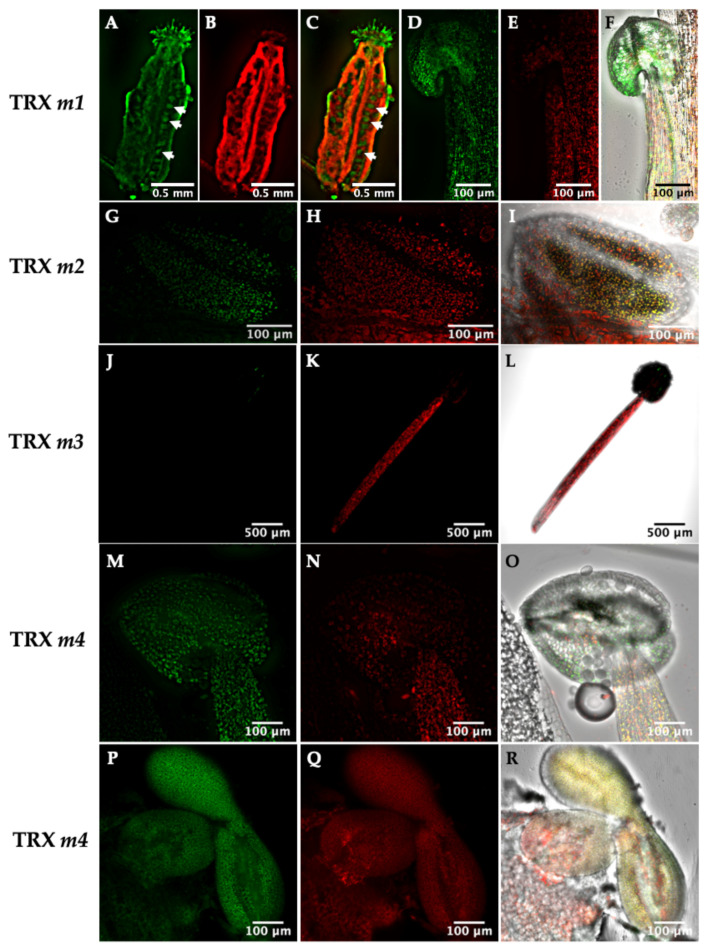
Representative confocal images of Arabidopsis lines expressing the TRX*m*::GFP constructs in reproductive tissues of 4-week-old Arabidopsis plants. TRX *m* expression was followed in different reproductive tissues: (**A**–**C**) ovary, (**D**–**O**) stamens and (**P**–**R**) torpedo stage embryo. Images show the emitted fluorescence signals or an overlay of the fluorescence and bright-field signals: fusion–protein fluorescence (**A**,**D**,**G**,**J**,**M**,**P**); chlorophyll autofluorescence (**B**,**E**,**H**,**K**,**N**,**Q**); computer overlay of the two fluorescence images (**C**); computer overlay of fluorescence and bright-field images (**F**,**I**,**L**,**O**,**R**). Arrows highlight ovules in an Arabidopsis ovary.

GFP-fused TRXs were also localized in reproductive organs such as flowers and embryos. In flowers, fluorescence was observed in stamens for TRXs *m1*, *m2* and *m4*; but not for TRX *m3* (Figure 3D–O). The GFP signal was more intense in the peripheral germ cells of anthers than in filaments for TRX*m2*::GFP (Figure 3G–I). We also detected the TRX*m1*::GFP fusion protein in ovules (arrows in Figure 3A–C). Interestingly, as they do not contain chlorophyll, there is no photosynthetic activity in the female gametophytes and TRX *m1* might be regulating light-independent processes. Regarding embryo localisation, a TRX*m4*::GFP signal localised at the late torpedo stage could be observed when chlorophyll accumulation begins in the forming cotyledons, co-localizing this pigment with the GFP signal (Figure 3P–R).

**Figure 4:**
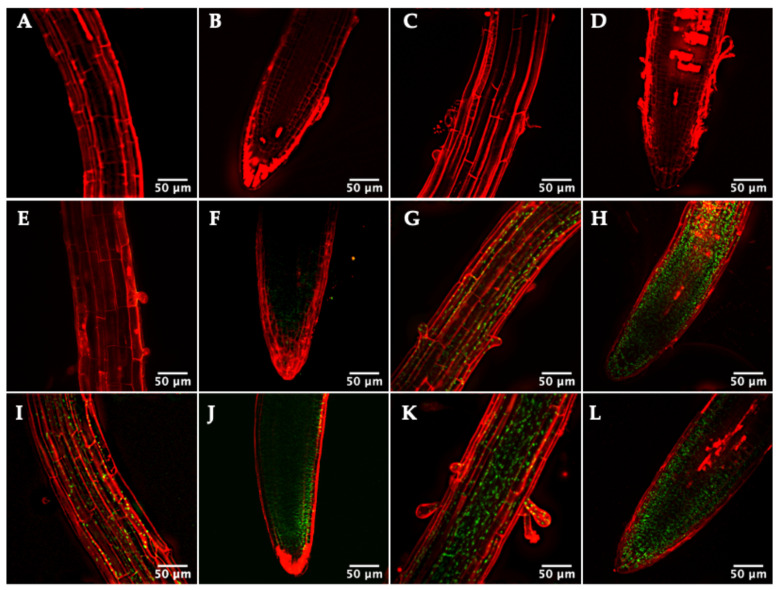
Representative confocal images of Arabidopsis lines expressing the TRX(*f/m*)::GFP constructs in roots of 7-day-old Arabidopsis seedlings. (**A**,**B**) TRX *f1*, (**C**,**D**) TRX *f2*, (**E**,**F**) TRX *m1*, (**G**,**H**) TRX *m2*, (**I**,**J**) TRX *m3*, (**K**,**L**) TRX *m4*. (**A**,**C**,**E**,**G**,**I**,**K**) Mature part of the root and (**B**,**D**,**F**,**H**,**J**,**L**) root meristem. Images show an overlay of GFP (green) and propidium iodide staining (red) fluorescences.

Remarkably, fluorescence in the TRX*m*::GFP lines was also detected in non-photosynthetic roots (Figure 4); however, no signal was detected in the TRX*f*::GFP lines (Figure 4A–D). GFP fluorescence was observed as sub-cellular spots inside cells, which were compatible with a plastid localization. Propidium iodide (PI) staining allowed the observation of the mature zone and apical region of roots by confocal microscopy. A TRX*m1*::GFP signal was found in the stele cells of the apical meristem, although the signal was weakly detected (Figure 4F). TRX*m2*::GFP fluorescence was strongly expressed in the apical meristem (Figure 4H) and in the cortex of the mature zone (Figure 4G). The root localization pattern of TRX*m3*::GFP (Figure 4I,J) was similar to that of TRX*m2*::GFP, although the distribution and intensity in the apical meristem were different. Finally, the intense signal of TRX*m4*::GFP stood out in the vascular cylinder and throughout the apical meristem (Figure 4K,L).

### 3.2. Photosynthesis, Stomatal Conductance and Transpiration of the TRX Mutants

**Figure 5:**
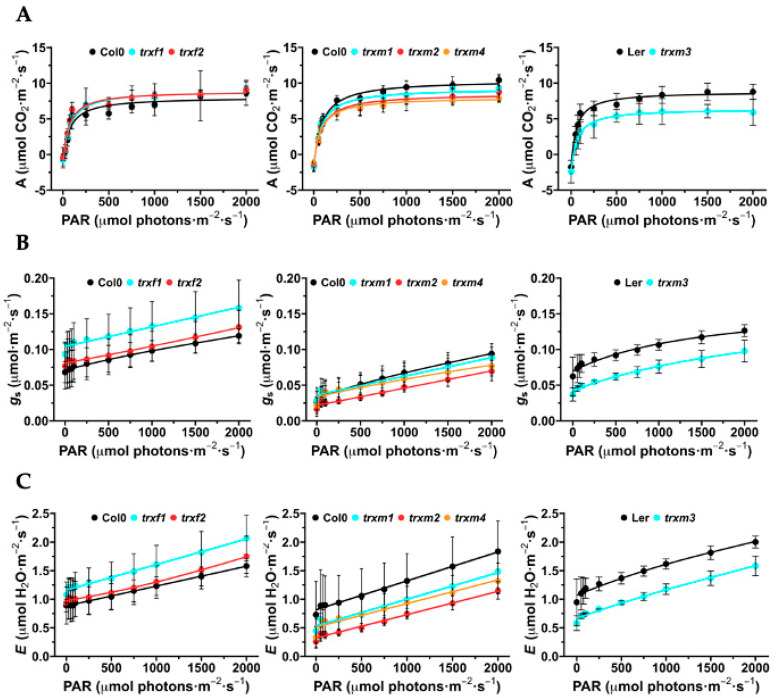
Infrared gas analyser measurements in WT (Col0 and Ler) and *trxf* and *trxm* mutant lines. (**A**) Light-response curves for the rate of photosynthetic CO_2_ assimilation (*A*); (**B**) stomatal conductance (*g*_s_); (**C**) transpiration (*E*). Arabidopsis plants were grown for 4 weeks.

Photosynthetic rate (*A*), transpiration (*E*) and stomatal conductance (*g*_s_) were measured on attached leaves of Arabidopsis loss-of-function lines *trxf1*, *trxf2*, *trxm1*, *trxm2*, *trxm3* and *trxm4* by using an open IRGA system (Figure 5).

Light response curves (*A*/*Q*) showed that wild type plants reached a maximum CO_2_ assimilation at 2000 µmol photons·m^−2^·s^−1^. We observed a similar photosynthetic rate throughout the light curve for *trxf1* in relation to Col0, which was slightly higher for *trxf2* (Figure 5A). The photosynthetic parameters *E* and *g*_s_ increased in parallel with light intensity, and the line *trxf1* had higher *E* and *g*_s_ values than those obtained for WT and *trxf2* (Figure 5B,C). At light intensities > 250 µmol photons·m^−2^·s^−1^, the mutant lines lacking TRX *m1*, *m2* and *m4* displayed lower values of the photosynthetic parameters (Figure 5A). Interestingly, *E* and *g*_s_ of *trxm2* were almost half that displayed by WT plants (Figure 5B,C). The CO_2_ assimilation rate and the *E* and *g*_s_ values of *trxm3* plants were clearly 25% lesser than its control Ler (Figure 5B,C), suggesting that, despite the low expression of TRX *m3* in the leaf, it would be exerting a noticeable control (direct or indirect) on photosynthesis.

### 3.3. Stomata Number and Aperture of the TRXs Mutants

**Figure 6:**
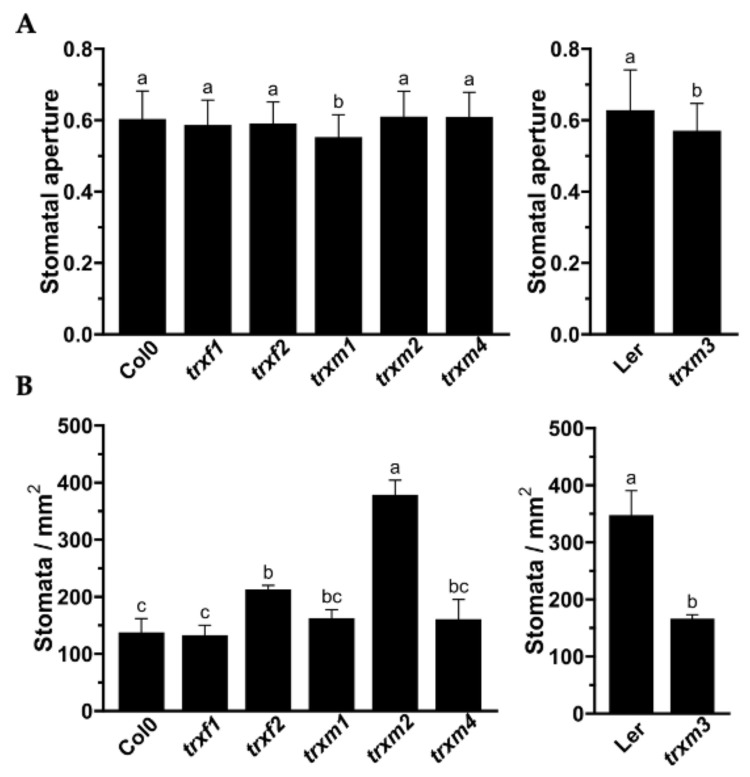
Stomatal characterization in WT (Col0 and Ler) and *trxf* and *trxm* mutant lines. (**A**) Stomatal aperture calculated as the width/length ratio. (**B**) Stomatal density of the adaxial leaf epidermis. Stomatal measurements were carried out with ImageJ 2.0.0. Different letters indicate a statistically significant difference (*p* < 0.05) according to a one-way analysis followed by a Tukey’s post hoc test. *n* = 50 per genotype.

Stomata are essential for leaf gas exchange and can influence the photosynthesis rate. Expression of fusion proteins TRX::GFP in stomata, together with the stomatal conductance measured in the Arabidopsis mutants, prompted us to carry out a stomatal characterization using scanning electron microscopy (SEM). Stomata number and aperture on the adaxial side of WT plants and *trxf* and *trxm* mutant lines are shown in Figure 6. With the exception of *trxf1*, mutant lines (*trxf2*: 210 stomata/mm^2^, *trxm1* and *trxm4*: ~160 stomata/mm^2^, *trxm2*: 378 stomata/mm^2^) had a higher number than WT Col0 (136 stomata/mm^2^) (Figure 6B). Conversely, the *trxm3* epidermis showed half the number of stomata (165 stomata/mm^2^) in comparison to WT Ler (346 stomata/mm^2^). Interestingly, in TRX *m2* deficient plants there were more than twice the number of stomata per unit area in comparison to WT Col0. In relation to stomata aperture (Figure 6A), no differences were detected among lines, with the exception of *trxm1* and *trxm3*, which showed slightly lower stomatal apertures in relation to their respective WT lines (10% and 7%, respectively).

### 3.4. Primary Root Structure of the TRX Mutants

**Figure 7:**
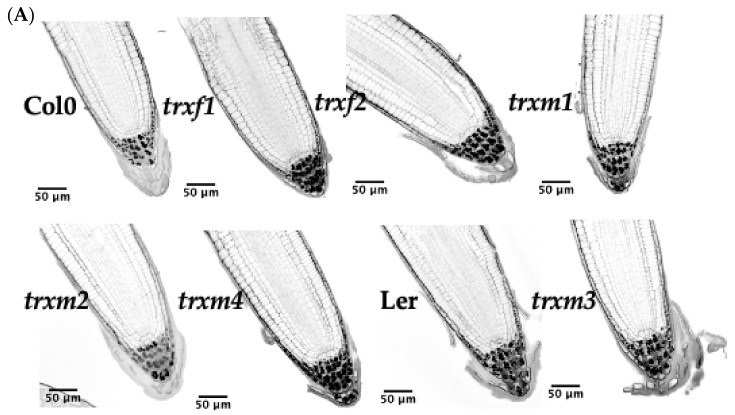

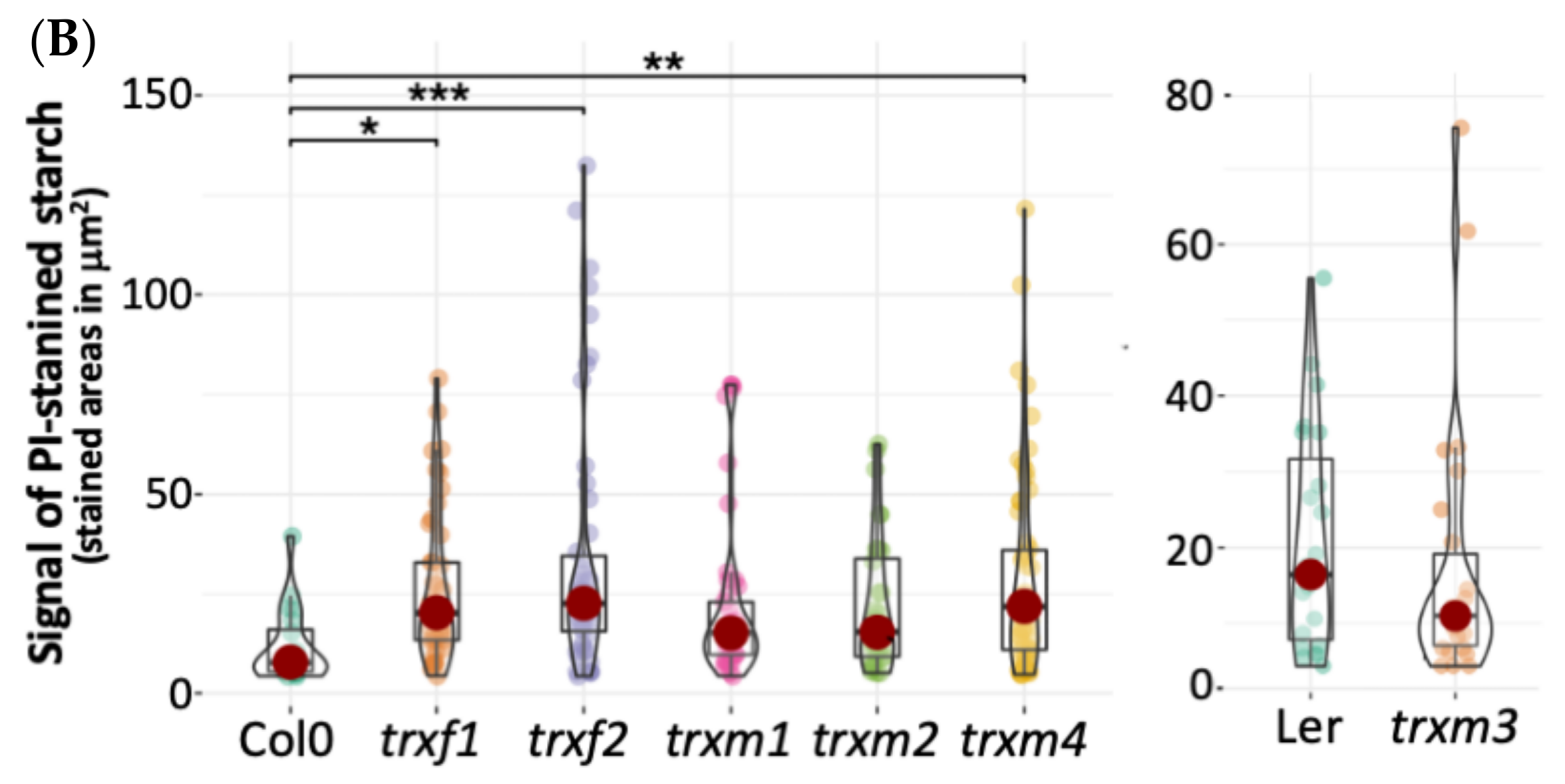
Root tip structure and starch signals in columella of WT (Col0 and Ler) and *trxf* and *trxm* mutant lines. (**A**) Root cells and starch granules were stained with propidium iodide (PI) and imaged with confocal microscopy. Arabidopsis seedlings were grown for 7 days. (**B**) Quantification of starch areas stained with PI (using the free software ImageJ 2.0.0). Asterisks above the violin graph indicate statistically significant differences (* *p* ≤ 0.05, ** *p* ≤ 0.01, *** *p* ≤ 0.001) as determined using the Kruskal–Wallis test and Dunn’s test.

Detecting the TRX *m*::GFP signal in roots led us to study the root phenotype in the TRX loss-of-function Arabidopsis mutants, extending our analyses to the knock-out *trxf* lines too. By staining the apical region of the roots with PI, we observed different structures such as the elongation area, the meristematic zone (xylem, phloem and pericycle cells) and the columella (Figure 7A). Starch granules increased in the columella of lines *trxf1*, *trxf2* and *trxm4*, decreased in *trxm3*, and were similar in *trxm1* and *trxm2* with respect to the WT line (Figure 7A,B). No significant differences were detected in the starch granules of the mutant line *trxm3* in relation to its control line Ler. Regarding root dimensions, *trxf2*, *trxf1, trxm4* and *trxm2* root widths were slightly higher than for Col0 (in the listed order), while the line *trxm1* showed a similar size. Additionally, the size of the columella of *trxf2* was apparently bigger than for Col0. Finally, the *trxm3* root tip was similar to its WT line (Figure 7).

### 3.5. Immunodetection of Plastid TRXs and Target Proteins in Leaves and Roots

**Figure 8:**
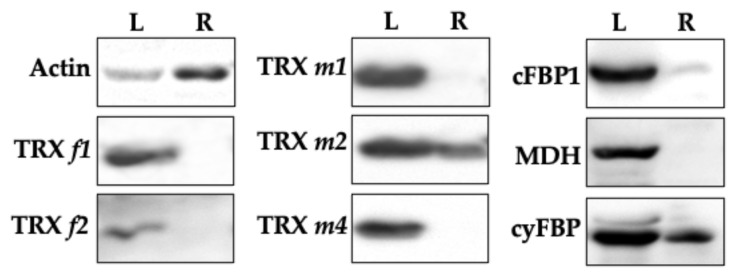
Immunodetection of TRXs *m* and *f* and targets cFBP1 and MDH in leaves and roots of Arabidopsis plants. L, leaves; R, roots. cyFBP (cytosolic FBPase) was included in the Western blotting analyses as a positive control of root expression. Tissues of 21-day-old plants were analysed.

**Figure 9:**
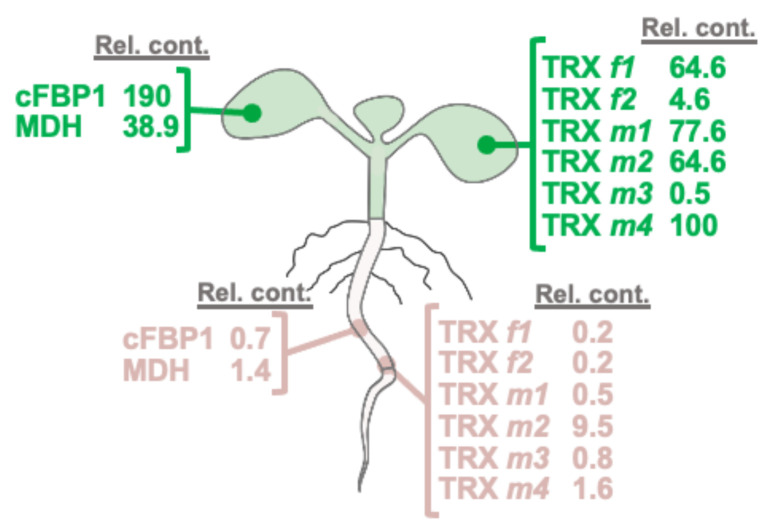
Protein relative content of plastid thioredoxins and their classical targets cFBP1 and MDH in cotyledons and roots of Arabidopsis. Presented values are relative to that of the most abundant TRX in cotyledons (TRX *m4*). Data were obtained from the database “Proteomics DB” (ProteomicsDB: a multi-omics and multi-organism resource for life science research. Available online: https://www.proteomicsdb.org/, accessed on 16 June 2022).

The expression level of TRXs *f* and *m* and their classical target proteins, cFBP1 and MDH, were determined by Western blotting by using specific antibodies in photosynthetic (leaves) and heterotrophic organs (roots). We also included in our analyses the cytosolic FBPase isoform (cyFBP) as a positive control for a protein expressed in roots [23]. Figure 8 shows that, as expected, TRXs *f* and *m*, cFBP1, cyFBP and MDH were present in leaves. Interestingly, TRX *m2* was significantly detected in roots, in a similar way to the positive control cyFBP. The rest of the TRXs could not be detected by the Western blotting technique. Remarkably, cFBP1 was also found in roots; nevertheless, we were not able to detect the MDH protein. As referred to in TRX *m* expression, the apparent inconsistency between the GFP and Western blotting results might be explained by the highest sensitivity of the fluorescence analyses. In fact, according to the database “Proteomics DB” (https://www.proteomics.db.org/, accessed on 16 June 2022), the most abundant *m*-type TRX in roots would be TRX *m2*, and the TRX *f* content would be 50 times lower (Figure 9). The rest of TRX *m*-type isoforms, though less expressed in roots than TRX *m2*, would be also more abundant than the *f*-type isoforms.

### 3.6. Functional Specificity of Arabidopsis TRXs f and m in the Redox Activation of FBPase

**Figure 10:**
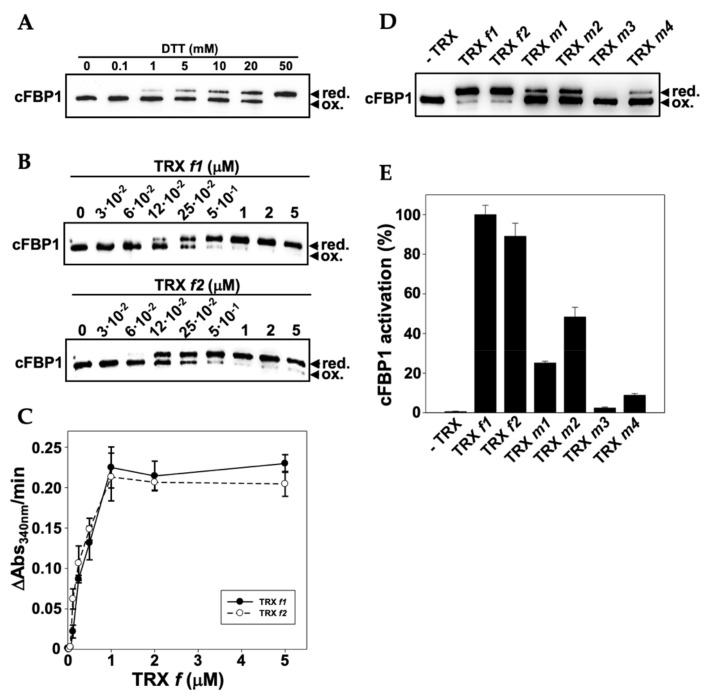
In vitro redox activation assays of cFBP1 by TRXs *f* and *m*. (**A**) Reduction assay and Western blotting experiment to determine the DTT concentration necessary to reduce cFBP1. (**B**,**C**) Western blotting experiments and FBPase activity assays, respectively, to calculate the TRX *f* concentrations needed to fully reduce cFBP1. Numbers in (**B**) show the TRX *f* concentrations (μM) used to activate cFBP1. (**D**,**E**) Western blotting experiments and FBPase activity assays, respectively, to determine TRXs *m* capacity, relative to TRX *f*, to reduce and activate cFBP1. Equal quantities of TRXs (1 μM) were used for cFBP1 activation. red., cFBP1 reduced; ox., cFBP1 oxidised. Three technical replicates were carried out for cFBP1 activity determinations (**C**,**E**).

TRX *f* efficiently activates cFBP1 and it is physiological reductant in leaf chloroplasts [29]. However, we did not detect TRX *f* in Arabidopsis roots (Figure 4 and Figure 8). One important question is how cFBP1 is then activated in this heterotrophic organ. In light of this, we carried out cFBP1 activation assays to check TRX *m* performance. Under our experimental conditions, a concentration of 0.1 mM of the reducing agent DTT was not enough to activate cFBP1 (Figure 10A). However, by adding 1 μM of TRX *f1* or *f2* to the reaction buffer, cFBP1 was completely reduced and reached the maximum activity (Figure 10B,C). By adding similar TRX *m* quantities to the reaction buffer, we obtained dissimilar cFBP1 activation levels (Figure 10D,E). TRX *m2* was the most efficient isoform reducing cFBP1, followed by TRX *m1*. In contrast, TRX *m3* displayed the poorest activating performance. All in all, our results (cFBP1 and TRX *m2* levels in roots and activity assays) strongly suggest that TRX *m2* might regulate cFBP1 activity in Arabidopsis roots. Additional in vivo interaction assays in roots would confirm the in vitro results.

## 4. Discussion

A remarkable feature of the TRX family is the diversity of isoforms found in Arabidopsis chloroplasts. Despite TRXs *f* and *m* being related to redox regulation of enzymes of C assimilation pathways, and therefore practically considered as photosynthetic proteins, works carried out in the last 15 years have shown that the expression of these redox players might also take place in non-photosynthetic organs [16,30,31]. Several studies have been aimed at understanding their roles in heterotrophic tissues. Among the first functions proposed were the activation of enzymes involved in the response to oxidative stress and hormone regulation in seeds [32]. However, information available is still poor. Because of a seeming overlap among different types and isoforms, it is challenging to distinguish between specificity and functional redundancy of plant TRXs. Moreover, the existence of other disulphide reductases that belong to the superfamily of TRXs, as is the case of GRXs or NTRC [33,34], makes it more difficult to understand how redox signalling is fine-tuning plant processes.

Among the TRXs present in chloroplasts, *f*- and *m*-types are the most diverse. In this work, we wanted to delve into the specific functions of these key redox proteins in photosynthetic and heterotrophic tissues. For this purpose, in order to know where these proteins are operating, we first carried out comprehensive expression analyses in Arabidopsis organs. As the average length promoter in Arabidopsis might be established at 500 bp [35], GFP translational fusions included a 1-kb DNA fragment upstream of the start codon. Regulatory sequences display putative binding motifs to transcription factors present in genes expressed in roots and flowers or involved in C metabolism, light regulation, seed and embryo development, and stress responses (data not shown). Confocal microscopy observations confirmed the functionality of the different translational fusions, as we expected GFP signals to be localized in chloroplasts. A rather surprising result was the observation of the TRX*f1*::GFP protein outside the chloroplast, not discarding the possibility of a dual localisation (probably in a smaller organelle). This result, although surprising, would support the experimental results previously obtained by Senkler and co-workers [36], which consisted of detecting TRX *f1* in mitochondria by proteomic analyses in Arabidopsis. The use of a specific fluorescence probe for mitochondria would confirm this alternative localisation. Curiously, Arabidopsis TRX *m2* is able to interact in vivo with the protein AtVDAC3 in mitochondria [37], pointing to a dual localisation for this *m*-type isoform. The presence of two isoforms of TRX *f* is not usual in plants, being, to date, the only known case in plants. This duplication in Arabidopsis (presumably TRX *f2*, due to its lower expression) might have started a specialization process, providing an adaptive advantage which would have helped to maintain a high selective pressure so that both isoforms can coexist.

As we also expected, the analyses of the TRX*m*::GFP translational fusions also established the presence of *m*-type TRXs in chloroplasts, but the different sub-organellar patterns that we observed were also noteworthy. The most interesting patterns corresponded to TRXs *m2* and *m4*. These proteins showed conspicuous signals in some chloroplasts, suggesting that they would be carrying out specialised functions in these organelles (Figure 1). In fact, the in vitro TRX*s*–cFBP1 interaction assays showed different activation performances, indicating distinct TRX-target docking for the *m*-type isoforms (Figure 10).

Taking into account the well-known connection between TRXs *f* and *m* and photosynthesis, it was not surprising to observe changes in the photosynthetic parameters. Nevertheless, light response curves reflected two different behaviour patterns. On one hand, *trxf* mutants had values of net photosynthesis, transpiration and stomatal conductance equal to or slightly higher than the Col0 control line (Figure 5). On the contrary, the *trxm* mutants presented lower values than those determined for the WT lines in the photosynthetic parameters. The decrease in photosynthesis performance observed in all *trxm* mutants would suggest that (i) there is no compensation phenomenon by other *m*-type isoforms (at least complete), and (ii) each isoform might be regulating a particular process within photosynthesis and CO_2_ assimilation.

In addition to the mesophyll chloroplasts, TRX *f* proteins were also observed in the chloroplasts of stomatal guard cells, as Barajas et al. detected previously in the pea epidermis [16]. We have reported an increase in stomatal conductance and transpiration in the Arabidopsis loss-of-function mutant *trxf1* (Figure 5). In this regard, the KcTRX *f* promoter of *Kandelia candel* is also expressed in stomatal guard cells [38]. Interestingly, KcTRX *f* over-expressing plants reduced their stomatal aperture through the enhancement of the K^+^ efflux under drought conditions, increasing the water-retaining capacity. Our results also corroborate the data published in a recent work [39]. In this work, authors identified TRXs *m1*, *m2* and *m4* in Arabidopsis guard cells and underlined the role of plastidial and cytosolic thiol reductases in the control of stomatal functioning. One of the roles attributed to TRX *f1* in Arabidopsis is the control of starch synthesis and degradation through the activation of the enzymes AGPase and BAM1, respectively [40,41]. According to the bibliography, starch turnover in guard cells is linked to stomatal opening [42], and photosynthesis and transpiration are important parameters related to stomatal function. Despite the fact that stomatal conductance and transpiration were higher in *trxf1*, no changes were detected in relation to stomata number or aperture (Figure 5). However, the greater number of stomata in *trxf2* might explain the slight increase of the photosynthetic parameters in this mutant (Figure 5 and Figure 6).

In addition to *f*-type TRXs, TRX *m* has also been localised in stomatal chloroplasts. An impairment in the TRX *m2* signalling provokes an important increase in stomata number on the adaxial side of the leaf, whereas plants deficient in TRX *m3* suffer a substantial decrease. Indeed, ~2.7 fold more stomata were counted in *trxm2* in relation to WT Col0, and it seems that one of the functions of TRX *m2* would be connected to stomata development rather than to the control of stomatal aperture. These results suggest that, regarding stomatal control, TRX *m2* and *m3* would be regulating different physiological issues. Moreover, aperture was only affected in the *trxm1* and *trxm3* Arabidopsis mutants. All in all, it seems that the loss of each *m*-type isoform has a negative effect on photosynthesis. This negative effect might also be due to an impairment of the stomatal functioning triggered by redox imbalances in guard cells, as is the case of the Arabidopsis lines lacking cytosolic/mitochondrial NADPH-dependent TRX reductases (NTRs) or GRXs and 2-Cys peroxiredoxins present in plastids [39]. Courteille and colleagues (2013) described the role of TRX *m4* on the regulation of the cycle electron flux, which might induce changes in CO_2_ assimilation [43]. However, it remains to be clarified whether the differences found in stomatal opening are a direct consequence of the impairment of redox signalling mediated by plastid TRXs in guard cells.

Through the observation of transgenic lines by confocal microscopy, we also detected *m*-type TRXs in inflorescences, such as in anthers of stamens, suggesting a role in reproduction. In this sense, the location of TRXs *f* and *m* in pollen, anthers, style and ovules of the pea has been previously described [16]. On the other hand, the expression of TRX *y1* and TRX *m3* in flower buds and of ACHT3 (an atypical TRX) in pollen have been also described in Arabidopsis [9,44,45]. The fact that TRX *m* was observed in anthers might suggest a role in Arabidopsis reproductive tissues. As the TRX*f*::GFP signal was not evident in anthers, it would seem reasonable to consider that only TRX *m* might have a specific role in pollen gametogenesis. During embryonic growth, TRX*m4*::GFP fluorescence was associated with regions with a high rate of division, indicating possible roles in embryogenesis, at least at the late developmental stages. The presence of TRXs *f* and *m* have already been described in pea seeds [16], as well as TRX *m* within wheat endosperm amyloplasts [17]. However, as far as we know, plastid TRXs had not been reported to be in Arabidopsis embryos, up till now. It is possible that *m*-type isoforms are related to cell proliferation taking place during embryogenesis.

Finally, the localisation of the *m*-type TRXs in roots is rather intriguing. TRX*m*::GFP signals were detected in other zones of intense cell division, such as the root meristem, but also in mature zones (Figure 4). According to the expression level, the role of TRX *m1* in roots would be modest, at least under non-stress situations. These proteins seem to be confined in organelles, probably plastids (amyloplasts in the columella cells of the root cap and leucoplasts in the rest of the root), because they are more difficult to identify than in leaves due to the absence of chlorophyll. Interestingly, our expression results agree with an in silico study of Arabidopsis thiol reductases [46]. In that work, by transcriptomic data mining, authors showed that the plastid TRXs analysed were well expressed in flowers, siliques and seeds. Regarding the roots, TRX *m2* would be the most expressed plastid TRX in this organ. Taking into account transcriptomic data, there would be a direct correlation between transcript and protein levels of plastid TRXs. This correlation would imply that the expression of TRXs *m* and *f* might be mainly controlled at the transcriptional level, validating also the TRXs::GFP constructs (size of promoter-regulatory sequence, fusion protein stability or TRX import to plastids).

In order to further the discovery of some functions of *m*-type TRXs in heterotrophic tissues, defective mutant lines were used for all the isoforms studied. Observations using the confocal microscope indicated that the loss of TRXs *f1*, *f2* and *m4* were apparently affecting the number of starch granules in the columella (Figure 7). In this regard, redox regulation mediated by TRX *h* in amyloplasts regulates root gravitropism in poplars [47]. Thus, it seems that the TRX family proteins would have an important physiological role in the columella of roots. Little is known about the role of TRX *m* in roots, apart from the regulation of symplastic trafficking by TRX *m3*, which seems to be crucial for meristem root maintenance in Arabidopsis [48]. Nonetheless, a more in-depth study of roots phenotype must be conducted, including expression analyses under stress situations. Interestingly, corroborating the GFP results, Western blotting analyses identified the presence of TRX *m2* in roots (Figure 8), suggesting the existence of unknown functions still to be discovered, at least for this *m*-type isoform.

The protein relative content in cotyledons of Arabidopsis of the TRX classical targets cFBP1 and MDH shows that cFBP1 is clearly associated with green tissues. Among TRXs, TRX *m4* displays the highest value (Figure 10). However, in roots, TRX *m2* is estimated to be expressed ~6- to ~50-fold more than the other TRXs analysed. It was surprising to detect cFBP1 in roots (Figure 9), an enzyme associated to photosynthetic plastids, leading to exciting questions about its role in this organ. According to “Proteomics DB” data, TRX *y1* is expressed in roots, while TRX *y2* is also expressed in leaves [9]. It is interesting to underline that the estimated abundance of TRX *m2* would be approximately 50 times higher than TRX *y2*, and around 100 times higher than TRX *y1* (data available at “Proteomics DB”).

Co-localisation in roots of TRX *m2* (the only plastid TRX immunodetected) and cFBP1 prompted us to study the activation capacity of TRX *m* for plastidial FBPase. The ability to reduce cFBP1 by the different isoforms of TRXs *f* and *m* showed that TRX *m2* was the most efficient *m*-type isoform, alongside the *f*-type. These results highlight TRX *m2* as one key player for redox regulation in plastid roots, where it might be the most abundant plastid TRX.

## 5. Conclusions

Despite the fact that, for many years, the functions of TRXs *f* and *m* were thought to be restricted to photosynthetic tissues, in this study, we have highlighted possible new functions in stomata and interesting localisations in heterotrophic tissues such as the stamen and roots. The presence of TRX *m* in roots would open new research lines on redox regulation in non-photosynthetic plastids. It is possible that TRX *m* could functionally substitute TRX *f* in roots, regulating enzymes such as cFBP1. Although we have described particular expression patterns in photosynthetic organs, the immunodetection of TRX *m2* in roots would represent one of the most differentiating features for *m*-type isoforms reported so far in the model plant *A. thaliana*.

## Data Availability

Data is contained within the article and supplementary material.

## Supplementary Materials

The following supporting information can be downloaded at: https://www.mdpi.com/article/10.3390/antiox11071365/s1, Table S1: Oligonucleotides used for TRX cloning.

## References

[1] Buchanan B.B., Schürmann P., Wolosiuk R.A., Jacquot J.P. (2002). The ferredoxin/thioredoxin system: From discovery to molecular structures and beyond. Photosynth. Res..

[2] Montrichard F., Alkhalfioui F., Yano H., Vensel W.H., Hurkman W.J., Buchanan B.B. (2009). Thioredoxin targets in plants: The first 30 years. J. Proteom..

[3] Gütle D.D., Roret T., Müller S.J., Couturier J., Lemaire S.D., Hecker A., Dhalleine T., Buchanan B.B., Reski R., Einsle O. (2016). Chloroplast FBPase and SBPase are thioredoxin-linked enzymes with similar architecture but different evolutionary histories. Proc. Natl. Acad. Sci. USA.

[4] Yokochi Y., Yoshida K., Hahn F., Miyagi A., Wakabayashi K.I., Kawai-Yamada M., Weber A., Hisabori T. (2021). Redox regulation of NADP-malate dehydrogenase is vital for land plants under fluctuating light environment. Proc. Natl. Acad. Sci. USA.

[5] The Arabidopsis Genome Initiative (2000). Analysis of the genome sequence of the flowering plant *Arabidopsis thaliana*. Nature.

[6] Serrato A.J., Fernández-Trijueque J., Barajas-López J.D., Chueca A., Sahrawy M. (2013). Plastid thioredoxins: A “one-for-all” redox-signaling system in plants. Front. Plant Sci..

[7] Sahrawy M., Hecht V., Lopez-Jaramillo J., Chueca A., Chartier Y., Meyer Y. (1996). Intron position as an evolutionary marker of thioredoxins and thioredoxin domains. J. Mol. Evol..

[8] Motohashi K., Kondoh A., Stumpp M.T., Hisabori T. (2001). Comprehensive survey of proteins targeted by chloroplast thioredoxin. Proc. Natl. Acad. Sci. USA.

[9] Collin V., Lamkemeyer P., Miginiac-Maslow M., Hirasawa M., Knaff D.B., Dietz K.-J., Issakidis-Bourguet E. (2004). Characterization of plastidial thioredoxins from Arabidopsis belonging to the new *y*-type. Plant Physiol..

[10] Chibani K., Wingsle G., Jacquot J.P., Gelhaye E., Rouhier N. (2009). Comparative genomic study of the thioredoxin family in photosynthetic organisms with emphasis on *Populus trichocarpa*. Mol. Plant.

[11] Fernández-Trijueque J., Serrato A.J., Sahrawy M. (2019). Proteomic analyses of thioredoxins *f* and *m Arabidopsis thaliana* mutants indicate specific functions for these proteins in plants. Antioxidants.

[12] Serrato A.J., Rojas-González J.A., Torres-Romero D., Vargas P., Mérida Á., Sahrawy M. (2021). Thioredoxins *m* are major players in the multifaceted light-adaptive response in *Arabidopsis thaliana*. Plant J..

[13] Vieira Dos Santos C., Rey P. (2006). Plant thioredoxins are key actors in the oxidative stress response. Trends Plant Sci..

[14] Meyer Y., Belin C., Delorme-Hinoux V., Reichheld J.P., Riondet C. (2012). Thioredoxin and glutaredoxin systems in plants: Molecular mechanisms, crosstalks, and functional significance. Antioxid. Redox Signal..

[15] Geigenberger P., Thormählen I., Daloso D.M., Fernie A.R. (2017). The unprecedented versatility of the plant thioredoxin system. Trends Plant Sci..

[16] Barajas-López J.D., Serrato A.J., Olmedilla A., Chueca A., Sahrawy M. (2007). Localization in roots and flowers of pea chloroplast thioredoxin *f* and *m* proteins reveals new roles in non-photosynthetic organs. Plant Physiol..

[17] Balmer Y., Vensel W.H., DuPont F.M., Buchanan B.B., Hurkman W.J. (2006). Proteome of amyloplasts isolated from developing wheat endosperm presents evidence of broad metabolic capability. J. Exp. Bot..

[18] Lawson T., Vialet-Chabrand S. (2016). Speedy stomata, photosynthesis and plant water use efficiency. New Phytol..

[19] Sasidharan R., Schippers J., Schmidt R.R. (2021). Redox and low-oxygen stress: Signal integration and interplay. Plant Physiol..

[20] Considine M.J., Foyer C.H. (2021). Oxygen and reactive oxygen species-dependent regulation of plant growth and development. Plant Physiol..

[21] Yoshida K., Hisabory T. (2016). Two distinct redox cascades cooperatively regulate chloroplast functions and sustain plant viability. Proc. Natl. Acad. Sci. USA.

[22] Serrato A.J., Romero-Puertas M.C., Lázaro-Payo A., Sahrawy M. (2018). Regulation by *S*-nitrosylation of the Calvin-Benson cycle fructose-1,6-bisphosphatase in Pisum sativum. Redox Biol..

[23] Nakagawa T., Kurose T., Hino T., Tanaka K., Kawamukai M., Niwa Y.K., Matsuoka K., Jinbo T., Kimura T. (2007). Development of series of gateway binary vectors, pGWBs for realizing efficient construction of fusion genes for plant transformation. J. Biosci. Bioeng..

[24] Heim R., Cubitt A.B., Tsien R.Y. (1995). Improved green fluorescence. Nature.

[25] Alassimone J., Naseer S., Geldner N. (2010). A developmental framework for endodermal differentiation and polarity. Proc. Natl. Acad. Sci. USA.

[26] Rojas-González J.A., Soto-Súarez M., García-Díaz A., Romero-Puertas M.C., Sandalio L.M., Mérida A., Thormählen I., Geigenberger P., Serrato A.J., Sahrawy M. (2015). Disruption of both chloroplastic and cytosolic FBPases genes results in dwarf phenotype and important starch and metabolite changes in *Arabidopsis thaliana*. J. Exp. Bot..

[27] Dong J., Chen W. (2013). The role of autophagy in chloroplast degradation and chlorophagy in immune defenses during Pst DC3000 (AvrRps4) infection. PLoS ONE.

[28] Wang Q., Sullivan R.W., Kight A., Henry R.L., Huang J., Jones A.M., Korth K.L. (2004). Deletion of the chloroplast-localized Thylakoid formation1 gene product in Arabidopsis leads to deficient thylakoid formation and variegated leaves. Plant Physiol..

[29] Ojeda V., Pérez-Ruiz J.M., González M., Nájera V.A., Sahrawy M., Serrato A.J., Geigenberger P., Cejudo F.J. (2017). NADPH thioredoxin teductase C and thioredoxins act concertedly in seedling development. Plant Physiol..

[30] Pagano E.A., Chueca A., López-Gorgé J. (2000). Expression of thioredoxins *f* and *m*, and of their targets fructose-1,6-bisphosphatase and NADP-malate dehydrogenase, in pea plants grown under normal and light/temperature stress conditions. J. Exp. Bot..

[31] Traverso J.A., Vignols F., Cazalis R., Serrato A.J., Pulido P., Sahrawy M., Meyer Y., Cejudo F.J., Chueca A. (2008). Immunocytochemical localization of *Pisum sativum* TRXs *f* and *m* in non-photosynthetic tissues. J. Exp. Bot..

[32] Née G., Châtel-Innocenti G., Meimoun P., Leymarie J., Montrichard F., Satour P., Bailly C., Issakidis-Bourguet E. (2021). A new role for plastid thioredoxins in seed physiology in relation to hormone regulation. Int. J. Mol. Sci..

[33] Serrato A.J., Pérez-Ruiz J.M., Spínola M.C., Cejudo F.J. (2004). A novel NADPH thioredoxin reductase, localized in the chloroplast, which deficiency causes hypersensitivity to abiotic stress in *Arabidopsis thaliana*. J. Biol. Chem..

[34] Meyer Y., Buchanan B.B., Vignols F., Reichheld J.P. (2009). Thioredoxins and glutaredoxins: Unifying elements in redox biology. Annu. Rev. Genet..

[35] Korkuc P., Schippers J.H., Walther D. (2014). Characterization and identification of cis-regulatory elements in Arabidopsis based on single-nucleotide polymorphism information. Plant Physiol..

[36] Senkler J., Senkler M., Eubel H., Hildebrandt T., Lengwenus C., Schertl P., Schwarzländer M., Wagner S., Wittig I., Braun H.P. (2017). The mitochondrial complexome of *Arabidopsis thaliana*. Plant J..

[37] Zhang M., Takano T., Liu S., Zhang X. (2015). Arabidopsis mitochondrial voltage-dependent anion channel 3 (AtVDAC3) protein interacts with thioredoxin *m2*. FEBS Lett..

[38] Jing X., Yao J., Ma X., Zhang Y., Sun Y., Xiang M., Hou P., Li N., Zhao R., Li J. (2020). *Kandelia candel* thioredoxin *f* confers osmotic stress tolerance in transgenic tobacco. Int. J. Mol. Sci..

[39] Montillet J.L., Rondet D., Brugière S., Henri P., Rumeau D., Reichheld J.P., Couté Y., Leonhardt N., Rey P. (2021). Plastidial and cytosolic thiol reductases participate in the control of stomatal functioning. Plant Cell Environ..

[40] Ballicora M.A., Frueauf J.B., Fu Y., Schürmann P., Preiss J. (2000). Activation of the potato tuber ADP-glucose pyrophosphorylase by thioredoxin. J. Biol. Chem..

[41] Valerio C., Costa A., Marri L., Issakidis-Bourguet E., Pupillo P., Trost P., Sparla F. (2011). Thioredoxin-regulated β-amylase (BAM1) triggers diurnal starch degradation in guard cells, and in mesophyll cells under osmotic stress. J. Exp. Bot..

[42] Santelia D., Lunn J.E. (2017). Transitory Starch Metabolism in Guard Cells: Unique Features for a Unique Function. Plant Physiol..

[43] Courteille A., Vesa S., Sanz-Barrio R., Cazale A.C.C., Becuwe-Linka N., Farran I., Havaux M., Rey P., Rumeau D. (2013). Thioredoxin *m4* controls photosynthetic alternative electron pathways in Arabidopsis. Plant Physiol..

[44] Mestres-Ortega D., Meyer Y. (1999). The Arabidopsis thaliana genome encodes at least four thioredoxins *m* and a new prokaryotic-like thioredoxin. Gene.

[45] Becker J.D., Boavida L.C., Carneiro J., Haury M., Feijó J.A. (2003). Transcriptional profiling of Arabidopsis tissues reveals the unique characteristics of the pollen transcriptome. Plant Physiol..

[46] Belin C., Bashandy T., Cela J., Delorme-Hinoux V., Riondet C., Reichheld J.P. (2015). A comprehensive study of thiol reduction gene expression under stress conditions in *Arabidopsis thaliana*. Plant Cell Environ..

[47] Azri W., Brunel N., Franchel J., Ben Rejeb I., Jacquot J.P., Julien J.L., Herbette S., Roeckel-Drevet P. (2013). Putative involvement of Thioredoxin *h* in early response to gravitropic stimulation of poplar stems. J. Plant Physiol..

[48] Benitez-Alfonso Y., Cilia M., San Roman A., Thomas C., Maule A., Hearn S., Jackson D. (2009). Control of Arabidopsis meristem development by thioredoxin-dependent regulation of intercellular transport. Proc. Natl. Acad. Sci. USA.

