# Supplementary material for: Comprehensive Expression Analyses of Plastidial Thioredoxins of Arabidopsis thaliana Indicate a Main Role of Thioredoxin m2 in Roots
Source: Antioxidants (Basel). 2022 Jul 14;11(7):1365. doi: 10.3390/antiox11071365 (PMC9311637; doi:10.3390/antiox11071365)
Supplement: Supplementary file 1 [file antioxidants-11-01365-s001.zip › antioxidants-1799035-supplementary.pdf]

**Table S1.** Oligonucleotides used for Arabidopsis TRXs cloning.

| Gene | Accession Number | Oligo Name | Sequence | Cloning |
|---|---|---|---|---|
| *TRXf1* | At3g02730 | f1ENT5PROM | CACCACTGTCTCCGCCTGAG | pGWB4 |
| | | f1ENT3 | TCCTCCGGAAGCAGCAGAC | |
| | | 5Atf1N | GGGGCATATGGTTGGTCAGGTGACGGAGGT | pET28b |
| | | 3Atf1B | GGGGGGATCCTCATCCGGAAGCAGCAGACCTCG | |
| *TRXf2* | At5g16400 | f2ENT5PROM | CACCAAACTCGAGTCTTTCAGTGC | pGWB4 |
| | | f2ENT3 | TCCGCCTGACCTTGCTGCTTC | |
| | | 5Atf2N | GGGGCATATGGTTGGCCAGGTGACGGAGGT | pET28b |
| | | 3Atf2B | GGGGGGATCCTCAGCCTGACCTTGCTGCTTCAA | |
| *TRXm1* | At1g03680 | m1ENT5PROM | CACCCCCTGTGATAGTTGTCACACAAG | pGWB4 |
| | | m1ENT3 | CAAGAATTTGTTGATGCTGGTTGC | |
| | | 5Atm1N | GGGGCATATGCACACTGCTACAGGAATTCCAGTGG | pET28b |
| | | 3Atm1B | GGGGGGATCCTCATTACAAGAATTTGTTGATGCTGG | |
| *TRXm2* | At4g03520 | m2ENT5PROM | CACCCAGCGAGCTAAAGTGACATC | pGWB4 |
| | | m2ENT3 | TGGCAAGAACTTGTCGAGGC | |
| | | 5Atm2N | GGGGCATATGGAAACTACTACCGATATTCAAGTGGTCA ATGA | pET28b |
| | | 3Atm2B | GGGGGGATCCCGAGATCTTACAGACTCCAC | |
| *TRXm3* | At2g15570 | m3ENT5PROM | CACCGTCCAATAATTTCCCCACGGAG | pGWB4 |
| | | m3ENT3 | TGAGTTCAAGACTCTTTCAATGGCG | |
| | | 5Atm3N | GGGGCATATGGCTGAAGTTACACAACGATC | pET28b |
| | | 3Atm3B | GGGGGGATCCTCATGAGTTCAAGACTCTTTCAA | |
| *TRXm4* | At3g15360 | m4ENT5PROM | CACCGGAGAGAGTAGGGAAAATGGAG | pGWB4 |
| | | m4ENT3 | CTCGACCAAGAATCTTTCTATAGTTTTCTCC | |
| | | 5Atm4N | GGGGCATATGGACACCACTGCCGCCGCCGT | pET28b |
| | | 3Atm4B | GGGGGGATCCTCACTCGACCAAGAATCTTTCTATAGTT TCT | |

For pGWB4 cloning, cDNAs were first cloned into the pENTR™ vector. Underlined sequences show restriction sites *Nde*I (CATATG) and *Bam*HI (GGATCC).
